# Estrogen- and Satiety State-Dependent Metabolic Lateralization in the Hypothalamus of Female Rats

**DOI:** 10.1371/journal.pone.0137462

**Published:** 2015-09-04

**Authors:** Istvan Toth, David S. Kiss, Gergely Jocsak, Virag Somogyi, Eva Toronyi, Tibor Bartha, Laszlo V. Frenyo, Tamas L. Horvath, Attila Zsarnovszky

**Affiliations:** 1 Department of Physiology and Biochemistry, Szent Istvan University Faculty of Veterinary Science, Budapest, Hungary; 2 Department of Transplantation and Surgery, Semmelweis University, Budapest, Hungary; 3 Division of Comparative Medicine, Yale University School of Medicine, New Haven, CT, United States of America; 4 Department of Animal Physiology and Animal Health, Szent Istvan University Faculty of Agricultural and Environmental Sciences, Godollo, Hungary; Hosptial Infantil Universitario Niño Jesús, CIBEROBN, SPAIN

## Abstract

Hypothalamus is the highest center and the main crossroad of numerous homeostatic regulatory pathways including reproduction and energy metabolism. Previous reports indicate that some of these functions may be driven by the synchronized but distinct functioning of the left and right hypothalamic sides. However, the nature of interplay between the hemispheres with regard to distinct hypothalamic functions is still unclear. Here we investigated the metabolic asymmetry between the left and right hypothalamic sides of ovariectomized female rats by measuring mitochondrial respiration rates, a parameter that reflects the intensity of cell and tissue metabolism. Ovariectomized (saline injected) and ovariectomized+estrogen injected animals were fed *ad libitum* or fasted to determine 1) the contribution of estrogen to metabolic asymmetry of hypothalamus; and 2) whether the hypothalamic asymmetry is modulated by the satiety state. Results show that estrogen-priming significantly increased both the proportion of animals with detected hypothalamic lateralization and the degree of metabolic difference between the hypothalamic sides causing a right-sided dominance during state 3 mitochondrial respiration (St3) in *ad libitum* fed animals. After 24 hours of fasting, lateralization in St3 values was clearly maintained; however, instead of the observed right-sided dominance that was detected in *ad libitum* fed animals here appeared in form of either right- or left-sidedness. In conclusion, our results revealed estrogen- and satiety state-dependent metabolic differences between the two hypothalamic hemispheres in female rats showing that the hypothalamic hemispheres drive the reproductive and satiety state related functions in an asymmetric manner.

## Introduction

It has been established for some time that the left and right sides of the nervous system are specialized to the regulation of certain specific, but distinct functions. Besides the well-known assignment of the two spinal cord sides in the regulation and/or mediation of left or right locomotor and autonomous functions, the functional lateralization of upper brain regions has also been recognized. For example, the functional asymmetry of the two cerebral hemispheres ensures the optimal integration of different cognitive processes, such as speech, spatial relation, fine motoric movements of hands, *etc*. Also, a considerable number of studies have found asymmetry in other brain areas such as the hippocampus, habenula, and thalamus (reviewed by [1–3]).

Asymmetry of the neuroendocrine hypothalamus, a center and crossroad of many homeostatic and reproductive regulatory functions, has also been indicated. For example, Cruz et al. [4] and Lopez et al. [5], found that effects of unilateral atropine (acetylcholine antagonist) and pilocarpine (acetylcholine agonist) implants into the preoptic-anterior hypothalamic areas were not only estrous phase-dependent but side-dependent as well. Even earlier, Gerendai et al. [6] found significantly more GnRH in one side of the hypothalamus than in the other. In line with those findings, experimental manipulation of the right side of the hypothalamus-gonad axis proved to be significantly more efficient than that of the left [7,8]. In addition to the afore-mentioned data, we have recently demonstrated that in normal cycling female rats, an estrous phase-dependent metabolic lateralization exists in the hypothalamus [9] that most likely plays an important and intimate role in the regulation of the (also asymmetric) ovarian cycle. It is also known that estrogen (E2) modulates mitochondrial activity in the hypothalamus [10], therefore it has been suggested that asymmetry in metabolic changes linked to reproductive functions is, at least in part, regulated by E2.

The hypothalamic regulation of reproduction and feeding is, on one hand, based on distinct hypothalamic morphological and biochemical bases, on the other hand, however, there is a well-known overlap between the afore-mentioned two regulatory circuits in the form of neuron populations and hormones involved in the regulation of both functions. Specifically, E2, besides coordinating female reproduction, also plays a key role in the regulation of appetite and energy expenditure as an anorexigenic factor [11–13]. Together with the existence of unilateral feeding pathways that connect hypothalamic structures to brain areas with asymmetric functions [14–16], this double role of E2 suggests that the hypothalamic regulation of food-intake may be just as lateralized as the regulation of reproductive functions.

The regulation of reproduction and food-intake, like all neuronal activities, is highly energy-dependent [17]. Therefore, mitochondrial ATP production is crucial in the supply of the hypothalamic energy needs and plays a permissive role in the regulation of the intensity of all energy consuming cellular processes. Thus, regulated mitochondrial respiration (terminal oxidation and oxidative phosphorylation) strongly correlates with the actual cellular energy consumption [18]. This idea offers the method of measuring mitochondrial respiration rates (*mrr*) to directly indicate the intensity (and changes in intensity) of overall functions in hypothalamic regions that are involved in the regulation of the afore-mentioned processes.

Besides of the female reproduction and food-intake regulation, asymmetry of other hypothalamic functions has also been indicated. For example, in male animals, Bakalkin et al. described left and right differences of GnRH levels in Wistar rats [19], while Inase and Machida found side-depended changes after unilateral orchiectomy in mice [20]. Furthermore, there are data about the asymmetry in the hypothalamic regulation of the cardiovascular system [21,22]; asymmetric distribution of thyroid-releasing hormone [23]; side-linked regulation of the circadian rhythm in the suprachiasmatic nucleus [24–26]; and lateralized functions has also been found in the central regulation of immune system [27,28]. These results further strengthen the idea of a complex mechanism within the hypothalamus, by which the two sides regulate the abundance of functions that are crammed into this relatively small brain area.

In summary, we propose that the hypothalamic asymmetry of female reproduction is estrogen-related, and asymmetry also applies to those functions that are partly or entirely involved in the hypothalamic regulation of feeding. In the present study, we examine the mitochondrial respiration rates, as a metabolic parameter, of the two hypothalamic sides in response to ovariectomy (saline injection) and ovariectomy+estrogen injection, in both conditions with respect to the satiety states of the animals.

## Experimental Procedures

### Animals

In the experiments, ten-week-old ovariectomized (*ovx*) female Wistar rats (*Rattus norvegicus*, breed: Crl:[WI]BR) were used 3 weeks after *ovx*. The study was conducted at the Faculty of Veterinary Sciences (Szent Istvan University, Hungary) in accordance with the Directive 2010/63/EU, and was approved by the Animal Health and Animal Welfare Directorate of the National Food Chain Safety Office (Permit Number: XIV-I-001/2202–4/2012).

The animals were obtained five weeks before the experiments (vendor: Semmelweis University, Basic Medical Science Center; Budapest, Hungary), and were kept in groups in controlled light (12-hour-long dark and light cycles; lights on at 7 a.m.). Regular rat chow (vendor: FarmerMix Kft., Zsambek, Hungary), and tap water were *ad libitum* available. After *ovx*, and in the experimental period, the animals were kept in groups of two. Before the experiment, the animals were randomly separated into two groups (n = 10), one of them remained *ad libitum* fed, while the other was fasted for 24 hours before sacrifice (quick guillotine decapitation at 7:00 a.m.) with constant water supply. Both groups were further divided to estrogen injected (17β-estradiol [E2], single dose of 23μg/100g body weight [BW], Sigma Aldrich Ltd., Hungary, water soluble), or sham injected (saline; S) subgroups. Since the most prominent effect on mitochondrial metabolism in the hypothalamus was registered between 8–10 hours after E2 treatment [10], subcutaneous injections were performed 10 hours before sacrifice (9 p.m. on the previous day). For estrogen substitution after *ovx*, water soluble estrogen is suggested due to the fact that it does not need specific binding protein for transportation [29].

In summary, the four experimental groups used are: estrogen treated *ad libitum* fed (E2+*ad lib*.; n = 5), estrogen treated fasted (E2+fasted; n = 6), sham injected *ad libitum* fed (S+*ad lib*.; n = 5), and sham injected fasted (S+fasted; n = 4) animals.

### Preparation of brain synaptosomal and perikaryal mitochondria and measurement of oxygen consumption

Mitochondrial fractions (containing both perikaryal and synaptosomal mitochondria) were obtained from the separated left and right hypothalamic sides (according to the findings by Toth et al. also termed as hemispheres [9]), then mitochondrial oxygen-consumption was measured. The method was reported in details by Toth et al. [9] (a detailed description can be also found in S1 Appendix), here, we only sum up the most important steps for general understanding. After quick guillotine decapitation, hypothalami were dissected as described earlier [30] then cut into left and right halves. Dissected brain samples were placed and further processed in ice-cold buffer starting from approximately 30 seconds after the decapitation. Hypothalamic samples were homogenized in isolation buffer, and mitochondria were purified by a Percoll gradient fractionation. As the last step of the separation procedure, the supernatant was poured off, and the pellet was stored on ice till the mitochondrial oxygen-consumption measurement. Samples were measured by a Clark-type oxygen electrode at 37°C. Measured values represent the mitochondrial respiration rate (*mrr*, given in consumed nmol O_2_ per ml of final volume in one minute). As the terminology of different mitochondrial respiration states varies in the relevant literature, we also explained the nomenclature in our previous study [9]. Shortly, we determined five states of mitochondrial respiration based on the respiration modifier added to the samples consecutively: mitochondrial oxygen consumption in respiration buffer only (no chemical added, state 1; St1); pyruvate and malate to fuel the Krebs’ cycle (state 2; St2); excess ADP (state 3; St3 or ADP-dependent respiration) oligomycin to block the ATP-synthase (state 4; St4); FCCP to deplete all remaining oxygen from the sample (state 5; St5). All respiration states lasted for 60 seconds.

### Data analysis

Although all mitochondrial respiration states were evaluated, St3 mitochondrial respiration *mrr* data were analyzed to determine functional sidedness since ADP/ATP ratio potently regulates mitochondrial activity [31]. The other mitochondrial respiration states were used as internal controls to monitor the suitability of the samples for analysis.

St3 *mrr* data were analyzed from two aspects. Firstly, we compared the left and right hypothalamic sides of individuals and the results gained from the comparison were used to shed light on the degree of the asymmetry, and to detect the more active side of the individual. We also compared the absolute *mrr* values collected from the active sides (as a data group) vs all less active sides (as another data group). This analysis of “population sidedness” highlights the metabolic activity itself on each side of the hypothalamus, instead of evaluating the differences between the two hemispheres. Thus, this approach provides insight into the case of metabolically balanced sides (i.e. minimal *mrr* differences), i.e., whether the metabolism of the two hemispheres are equally high or equally low under a given experimental conditions.

Because of the internal error of the described protocol (*cca* 10% in St3 in case of a 30mg hypothalamic block), we set up a strict requirement, and sidedness as a term was only used if the difference between left and right hypothalamic sides of the individual was 20% or higher.

Statistical analyses were conducted with the contribution of the Department of Biomathematics, Szent Istvan University Faculty at Veterinary Sciences using Fisher’s exact test (to determine sidedness), and two-way ANOVA with Bonferroni posttests (to analyze variance between groups) by Prism 5 (GraphPad Software Inc., San Diego, CA).

## Results and Discussion

The first findings that indicated hypothalamic sidedness were published more than 40 years ago (in the 1970’s); however, those studies seem to have been discontinued and hence, the exact nature and function of this phenomenon is still unknown. In a quest to further elucidate our previous finding that there is an estrous phase-dependent metabolic lateralization in the hypothalamus of female rats, and considering the anatomical and functional complexity of the neuroendocrine hypothalamus, here we demonstrated that the function of hypothalamic feeding centers is also lateralized and that this lateralization is influenced by E2.

In our experiments, 23μg/100g BW estradiol was administered subcutaneously based on the results of Kiss et al. [10]. They found that in this dose, a strong metabolic effect is developed shortly after the injection, and it has its peak in the mediobasal and lateral hypothalamus around 10 hours. In the present study, we tried to clarify the role of the estrogen in the earlier described estrous phase-depended hypothalamic sidedness, and it seemed to be reasonable to sacrifice the animals 10 hours after injection when the mitochondrial activity is the highest. The main consequence of this approach is that the detected effects on cellular metabolism are not the classical genomic response to estrogen, but they were most likely mediated by G protein coupled membrane receptors and/or they were direct effects on mitochondria [32].

**Fig 1:**
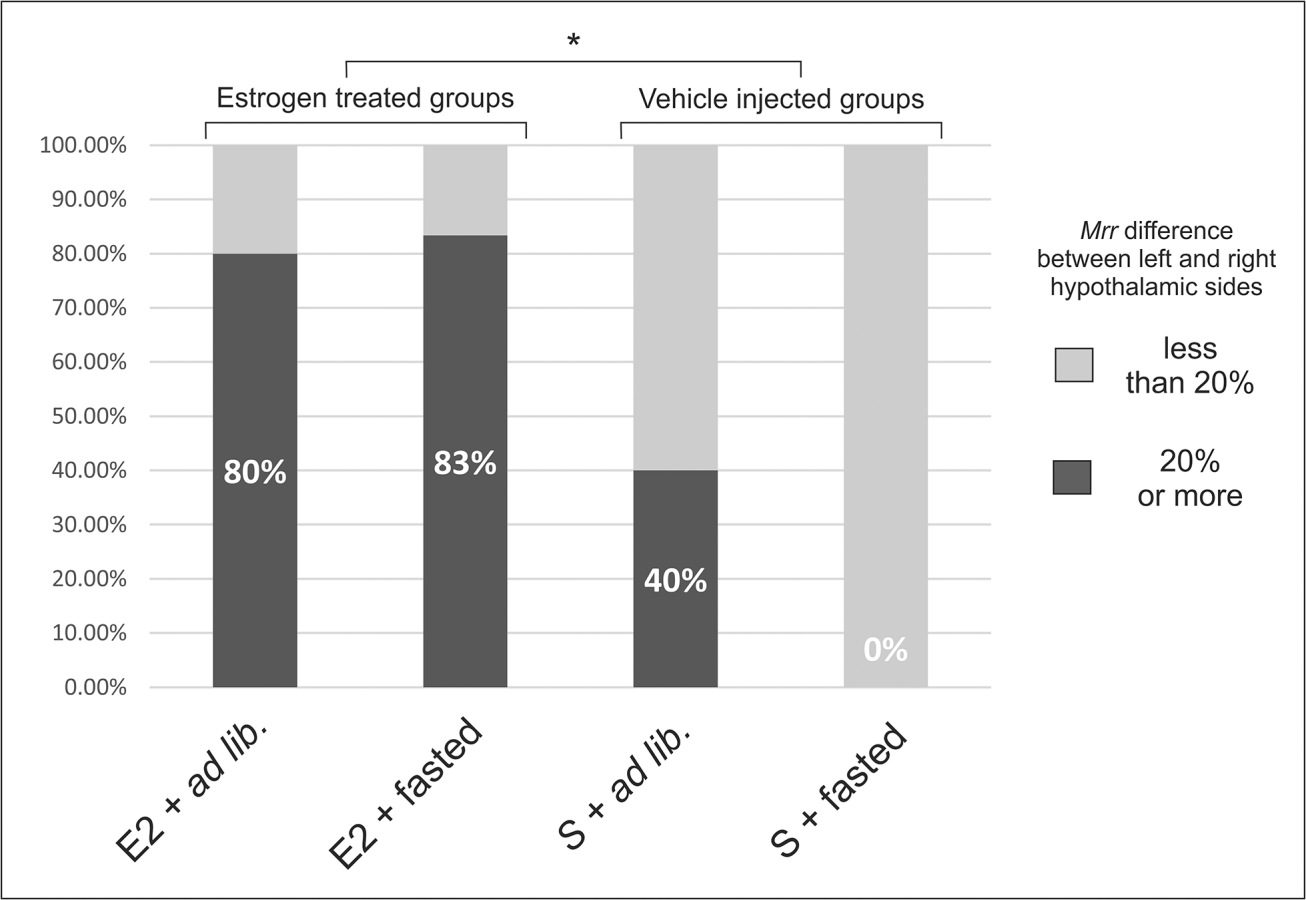
Percentage of animals with hypothalamic asymmetry in state 3 mitochondrial respiration rate (*mrr*). E2 treatment, regardless of satiety states, caused significantly higher proportion of sided animals (Fisher’s exact test, *: p<0.05). In absence of E2, 24 hours food restriction further reduced the extent of hypothalamic asymmetry. Experimental groups: E2+*ad lib*: estrogen treated, *ad libitum* fed animals (n = 5); E2+fasted: estrogen treated, fasted animals (n = 6); S+*ad lib*: vehicle injected, *ad libitum* fed animals (n = 5); S+fasted: vehicle injected, fasted animals (n = 4). Sidedness was considered if the difference between the left and right hypothalamic sides of the individual was 20% or higher.

Firstly, we determined the proportion of animals with metabolic sidedness of hypothalamus in each groups (left-right difference > 20%; Fig 1). This comparison yields basic information about how reproductive and satiety states influence the metabolic conditions in the two sides of hypothalamus. The most prominent result is that E2 treatment, regardless of satiety states, caused remarkably higher proportion of sided animals (Fisher’s exact test, p<0.05). This means that after bilateral ovariectomy, we could observe a hypothalamic state of low metabolic asymmetry in which the left and right sides’ capability to react to E2 seem to significantly differ. Similar effect have been described on the asymmetric GnRH levels by Gerendai et al. [6] suggesting that the underlying mechanisms of GnRH secretion may be modulated by the E2-related unilateral metabolic responses.

**Fig 2:**
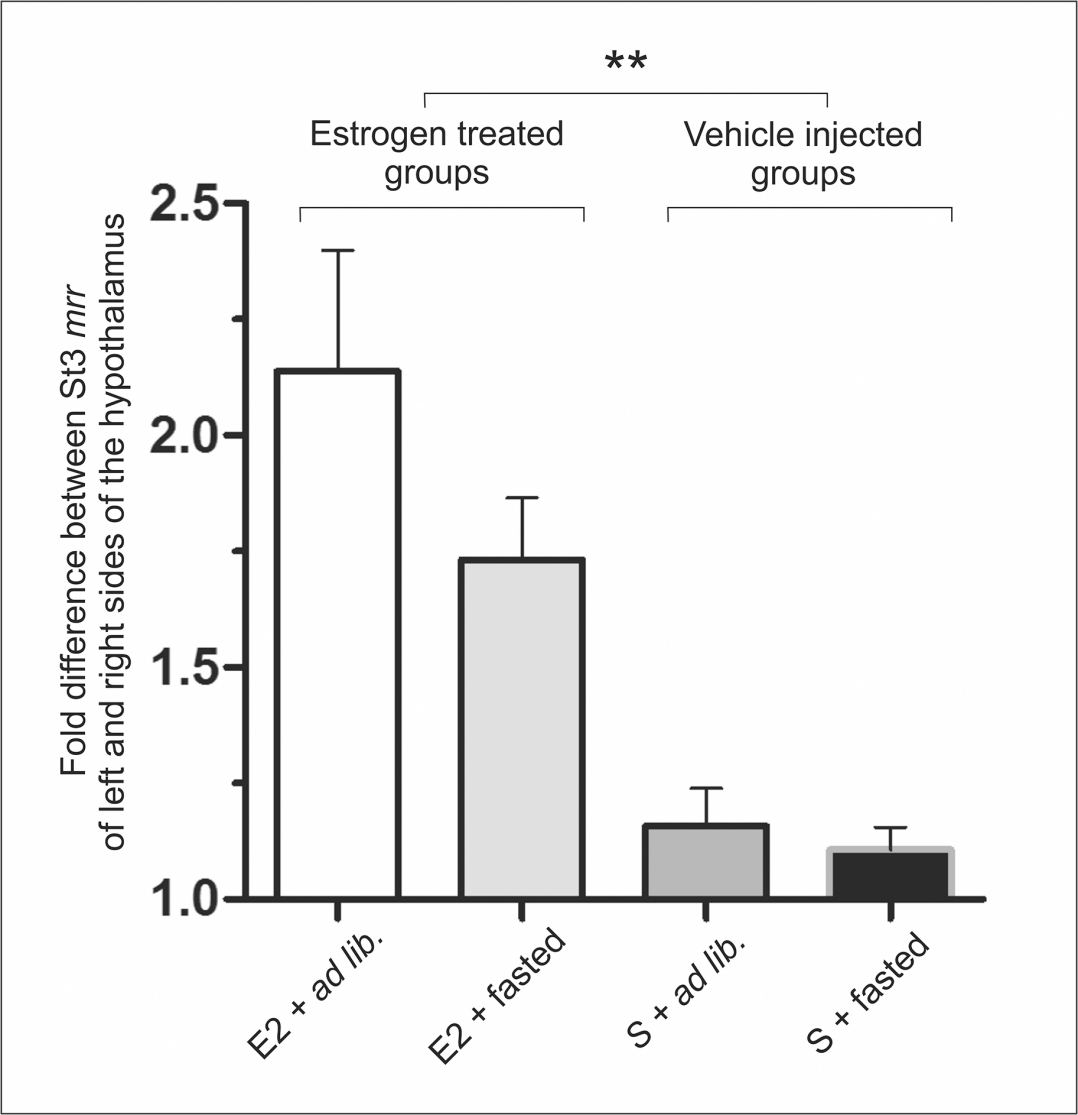
Degree of hypothalamic asymmetry depending on reproductive and satiety states. The presence of high E2 levels combined with *ad libitum* feeding provoked the most striking sidedness; E2+fasted animals show a little less remarkable sidedness; and gonadectomy (sham injection) nearly abolishes the differences between hypothalamic hemispheres (vehicle injected groups). Experimental groups: E2+*ad lib*: estrogen treated, *ad libitum* fed animals (n = 5); E2+fasted: estrogen treated, fasted animals (n = 6); S+*ad lib*: vehicle injected, *ad libitum* fed animals (n = 5); S+fasted: vehicle injected, fasted animals (n = 4). (Two-way ANOVA with Bonferroni posttests. E2 effects: ** p<0.01).

Further analyzing the data, we also examined the fold differences between left and right hypothalamic sides of individuals in order to see whether the higher proportion of sided animals in the estrogen treated groups is accompanied by a higher degree of asymmetry between the sides or the same difference can be observed in every sided animals (Fig 2). This analysis could fortify the role of E2 (two-way ANOVA: p<0.01), furthermore, it revealed some modifying effect of the satiety state of the animal. In summary, the presence of high E2 levels combined with *ad libitum* feeding provoked the most striking sidedness (E2+*ad lib*. group); E2+fasted animals showed a somewhat less remarkable sidedness, while gonadectomy (sham injection) nearly abolished the differences between hypothalamic hemispheres (vehicle injected groups). Interestingly, in *ad libitum* fed groups, the higher *mrr* values were mostly detected on right hypothalamic side of the individuals, while in the fasted groups, the number of left sided and right sided animals were balanced (data not shown).

By the single dose E2 injection of *ovx* animals, we intended to mimic the acute rise of estrogen concentration during the estrous cycle. As a result, we found early proestrus-like metabolic changes, supporting our earlier conclusion that the previously described estrous phase-dependent metabolic asymmetry is linked, at least in part, to the fluctuation of the plasma estrogen levels [9]. Other reports that focused on the rapid behavioral and endocrinological changes associated with the process of ovulation are also consonant with these observations [6,33–36]. Although extensive studies are needed to clarify the possible anatomical and biochemical bases of the hypothalamic functional asymmetry, one of the likely candidates that potentially mediate these E2 effects is the regulated expression of estrogen receptor alpha (ERα) that is known to rise earlier on the right side of preoptic and anterior hypothalamic areas [34]. While the phenomenon of the metabolic asymmetry seems to lie on the grounds of side-linked unequal potentials within the hypothalamus, further studies are needed to clarify whether it is based on genomic or non-genomic differences between the hypothalamic hemispheres. Moreover, since an overall sidedness exists in the female reproductive system [8,37], the putative role of genetic coding in sidedness should be investigated outside of the hypothalamus as well.

Taken together the afore-mentioned results regarding the role of estrogen in reproductive functions, we found strongly estrogen-dependent asymmetric metabolic changes in the hypothalamus that might well be attributed to estrogen driven rapid events leading to short term neuronal plasticity related to GnRH peak [38,39]. This idea is supported by findings showing that rapid and non-genomic estrogen effects include alterations in mitochondrial structure and function (reviewed by [40]) such us ATP synthesis [41,42].

**Fig 3:**
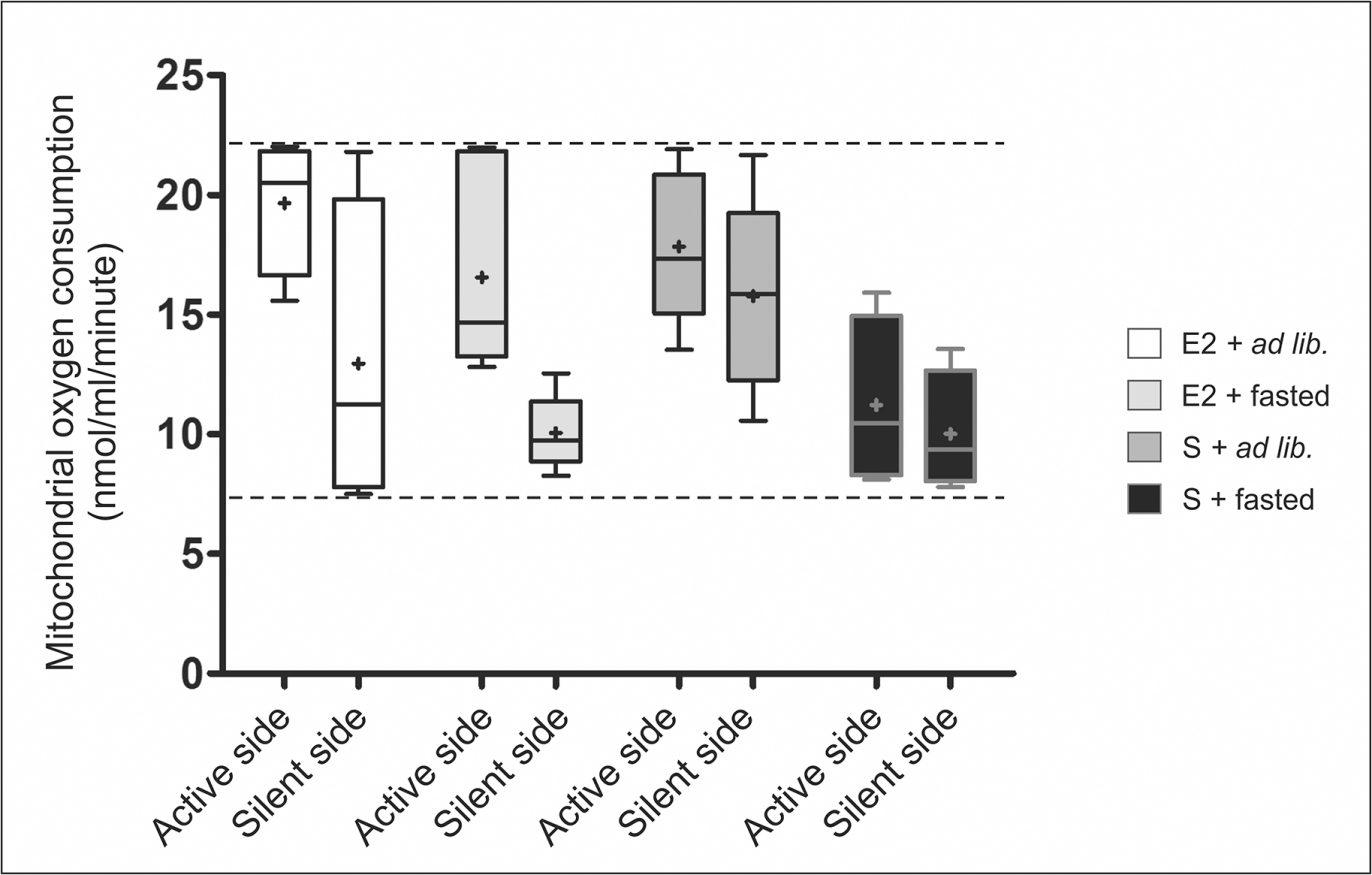
Boxplot diagram of “population sidedness” in state 3 mitochondrial respiration comparing the less active hypothalamic sides (silent sides) to the more active hypothalamic sides (active sides). E2 treatment causes high differences between the silent and active sides; whereas gonadectomy reduces the differences between the sides. 24 hours of fasting lowered the *mrr* values that could be increased unilaterally by single E2 injection (E2+fasted group). It seems that under our experimental circumstances, hypothalamic halves have a minimal and maximal activity (indicated with the broken lines). Experimental groups: E2+*ad lib*: estrogen treated, *ad libitum* fed animals; E2+fasted: estrogen treated, fasted animals; S+*ad lib*: vehicle injected, *ad libitum* fed animals; S+fasted: vehicle injected, fasted animals.

As the last aspect of our analysis (Fig 3), we also compared absolute *mrr* values collected from the more active sides (as a data group; in this sense dominant sides) vs all less active sides (as another data group). The comparison reinforces our findings showing that E2 treatment causes the highest sidedness, whereas gonadectomy reduces the differences between the sides. Furthermore, 24 hours of fasting lead to lowered *mrr* values; and also, as a subsidiary, albeit interesting outcome is that mitochondrial oxygen consumption rates are constrained into a range of a minimal (around 8nmol/ml/minute) and maximal (around 22nmol/ml/minute) activity regardless of experimental conditions. Taken into consideration the corresponding data of normal cycling female rats (data not shown; [9]), where this range falls between 8 and 50nmol/ml/minute, ovariectomy causes a drastic decline in the metabolic range that remains irreversible even by estrogen treatment. Detailed interpretation of this result, however, does not fit the scope of this study.

Although we have already described the estrous phase-dependent metabolic sidedness in normal cycling female rats [9], the present results brought at least two interesting additions to those observations: i) while sidedness in late proestrus and estrus was linked exclusively to the right side of the hypothalamus, without intact ovaries and after a single injection of E2 left sidedness could also develop; ii) the absence of the masking effect of E2, we found that 24 hours of food restriction reduced the proportion (number) of animals with sided hypothalamic metabolic intensities (Figs 1 and 2). These phenomena may be explained by considering that full reproductive effects of E2 are only exerted in the presence of all other ovarian hormones (i.e. progesterone, etc.), and without those hormones the anorexigenic effects of E2 [11,12] may develop more prominently (satiety hormone-like effects are more dominant). In summary, the afore-mentioned results, together with the existence of unilateral feeding pathways connecting hypothalamic structures to asymmetric brain areas [14–16], further support the idea that the food-intake related hypothalamic functions show a lateralized distribution between the two hypothalamic hemispheres.

As stated above, 24 hours of food deprivation led to lowered *mrr* values. This obviously raises questions if one considers the recently accepted concept stating that food deprivation enhances metabolic activity (i.e. mitochondrial ATP synthesis) of those neuron populations in the medial part of hypothalamus (nucleus arcuatus) that are prominently involved in the regulation of food-intake, as it was published by Cakir et al. [43]. It is to note, however, that they worked exclusively on male rats, and no female was included in their experiments. This data raises the possibility that food deprivation alters the mitochondrial activity of medial hypothalamus in different ways between sexes. Therefore, since, according to our present knowledge similar data are not yet available neither on female rats, nor in the relation of the lateral hypothalamus, understanding of our data requires region-specific if not neuron-specific investigations of mitochondrial responses on the applied conditions.

An obvious shortcoming of the metabolic screening method that we used is that hypothalamic sidedness was determined *post mortem*. Therefore, measurements cannot be repeated in the same individual nor the data can be used for the prediction of the exact type (right or left) of sidedness. We believe that further improvement of the technical methods to determine hypothalamic sidedness *in vivo* (i.e., the adoption of functional MRI or other methods) will help find answers to a plethora of questions that arise from the matter of hypothalamic asymmetry (such as whether or not the hypothalamic hemispheres are able to overtake each other’s functions), and would definitely help in the understanding of some forms of medical conditions with hypothalamic origin.

## Conclusion

Our results revealed estrogen- and satiety state-dependent metabolic differences between the two hypothalamic hemispheres in female rats. In the regulation of reproduction, it seems, a predetermined, strongly estrogen-related sidedness exists with a right sided dominance. On the other hand, hypothalamic regulation of feeding raises more difficulties: asymmetric, side-linked mechanisms seem to drive the feeding circuitries as well, but up-to-date neither us nor other studies (to our knowledge) could indicate the dominant hypothalamic side with regard to the food-intake regulation. Improved methodical approach and/or exactly pinpointed experimental conditions may henceforward clarify the physiological role of the distribution of tasks that remained hitherto veiled.

This study changes our current view on the regulation of female reproduction and food-intake, and may provide new perspectives for better understanding of these hypothalamic driven physiological processes. Also, disturbances of lateralized functions may take part in the pathogenesis of hypothalamus-linked health conditions, as it is already indicated in case of other brain areas [44].

## Supporting Information

S1 Appendix Fully detailed methods and material section, flow charts are included for easier understanding. (PDF)
