# Supplementary material for: Estrogen- and Satiety State-Dependent Metabolic Lateralization in the Hypothalamus of Female Rats
Source: PLoS One. 2015 Sep 4;10(9):e0137462. doi: 10.1371/journal.pone.0137462 (PMC4560379; doi:10.1371/journal.pone.0137462)
Supplement: S1 Appendix — (PDF) [file pone.0137462.s001.pdf]

Appendix

Reagents and equipment

I. Reagents

BSA	bovine serum albumin; fatty acid free (!); Sigma, cat. no. A7511
DMSO	dimethyl sulfoxide; Sigma, cat. no. D2650
EGTA	ethylene glycol-bis (2-aminoethylether)-N,N,N',N'-tetraacetic acid, $C_{14}H_{24}N_2O_{10}$; RNA and DNA free (!); Sigma, cat. no. E3889
Ethanol	C_2H_6O; pure for molecular biology; Sigma, cat. no. E7023
HEPES	HEPES potassium salt, 4-(2-Hydroxyethyl)piperazine-1-ethanesulfonic acidpotassium salt, $C_8H_{17}KN_2O_4S$; Sigma, cat. no. H0527
HCl	hydrochloric acid 37%; Carlo Erba, cat. no. 403871
KCL	potassium chloride; Sigma, cat. no. P5405
KH_2PO_4	potassium phosphate monobasic, H_2KO_4P; Sigma, cat. no. P5655
KOH	potassium hydroxide; Sigma, cat. no. P1767
Mannitol	D-Mannitol, $C_6H_{14}O_6$; Sigma, cat. no. M9546
$MgCl_2$	magnesium chloride solution; Sigma, cat. no. M1028
Na2S2O4	sodium dithionite; Sigma, cat. no. 452882
Sucrose	α-D-glucopyranosyl β-D-fructofuranoside; $C_{12}H_{22}O_{11}$; Sigma, cat. no. S7903
Percoll	GE Healthcare, cat. no. 17-0891-01

II. Buffer solutions

To set pH 7.2 use the 5M KOH and the 37% HCl solutions. These buffers should be stored at 4°C for not more than a couple days.

Isolation buffer with EGTA	Isolation buffer without EGTA	Respiration buffer
215mM Mannitol	215mM Mannitol	215mM Mannitol
75mM Sucrose	75mM Sucrose	75mM Sucrose
0.1% BSA	0.1% BSA	0.1% BSA

1mM EGTA	20mM HEPES	2mM MgCl
20mM HEPES		2.5mM KH_2PO_4
		20mM HEPES

III. Equipment

Filtration apparatus for preparation of Percoll solution with 90mm diameter (Millipore, Billerica, MA, USA; cat. no. AP1509000)

pH meter (SevenEasy S20; Mettler Toledo; Schwerzenbach, Switzerland)

Rodent guillotine (DCAP; Kent Scientific, Kent, UK)

Brain matrix rat 175-300g, 0.5mm coronal, stainless steel; (World Precision Instruments, Sarasota, FL, USA; cat. no. RBMS-300C)

10-15ml teflon-glass tissue grinder of type Potter-Elvehjem with a motor driven (used at 800rpm) pestle of 0.1–0.15mm clearance. (Wheaton Industries Inc.; Millville, NJ, USA)

2.0ml transparent conical microcentrifuge tubes for Percoll gradient containing steps (Eppendorf tubes, natural; Deltalab, Barcelona, Spain; cat. no. 4092.6N)

Bench-top centrifuge applied with a rotor no. 1689 (Hettich Universal 32 centrifuge; Hettich Instruments, Beverly, MA, USA; rotor: 30 sleeve, fixed 45°)

Clark-type oxygen electrode (OxygraphPlus oxygen electrode system, Hansatech Instruments, Norfolk, UK)

IV. Stocks of respiratory modifiers

ADP	adenosine 5′-diphosphate monopotassium salt dihydrate,<br>(128.2mg dissolved in 10ml distilled water [DW] + 200µl 1M HEPES)<br>$C_{10}H_{14}KN_5O_{10}P_2 \cdot 2H_2O$; FW: 501.32g/mol; Sigma, cat. no. A5285
FCCP	carbonyl cyanide 4-(trifluoromethoxy)phenylhydrazone,<br>(2.54mg dissolved in 10ml 100% DMSO)<br>$C_{10}H_5F_3N_4O$; FW: 254.17g/mol Santa Cruz, cat. no. sc-203578
Malate	L-(−)-malic acid,<br>(670.5mg malic acid dissolved in 10ml DW + 200µl 1M HEPES)<br>$C_4H_6O_5$; FW: 134.09g/mol; Sigma, cat. no. M7397
Oligomycin	oligomycin A,<br>1mg dissolved in 1ml ethanol<br>$C_{45}H_{74}O_{11}$; FW: 791.06g/mol; Santa Cruz, cat. no. sc-201551
Pyruvate	sodium pyruvate,<br>(550mg sodium pyruvate dissolved in 10ml DW + 200µl 1M HEPES)<br>$C_3H_3NaO_3$; FW: 110.04g/mol; Sigma, cat. no. P2256

ADP, malate and pyruvate should be fresh made on the day of the experiment while FCCP and oligomycin can be made up two several months and stored at -20 °C before use.

Detailed protocol

1. Dissection and tissue homogenization

The experimental design is also summed up on Fig. 1.

After quick guillotine decapitation, the hypothalamus was extracted in ice-cold environment as follows. After removing the skin and muscles, the skull was opened as described earlier [1], and the cranial part of the brain was slightly lifted with a cold spatula in order to cut the optic nerve. After cutting, the brain was gently removed, and placed into an ice-cold brain matrix. The connecting tissue from the basal part of the hypothalamus was removed with a fine forceps. Then, vertical incisions were made using ice-cold blades, for a coronal section of the entire hypothalamus: an incision right behind the rostral part of the *chiasma opticum* (Bregma -0.25), and an other one through the *corpus mamillare* (Bregma -5.0). The coronal sections were placed on the rostral surface, and the *piriform* and *entorhinal cortex*, then the thalamic area dorsal of the *fornices* were cut off. Finally, the hypothalamus was cut into left and right sides along the 3rd ventricle. The tissue blocks (30-35mg) were put into 750μl ice-cold isolation buffer, and stored until homogenization. Dissected brain samples were placed and further processed in ice-cold buffer starting from approximately 30 seconds after the decapitation.

The homogenization was performed in a motorized teflon-on-glass tissue homogenizer (Potter-Elvehjem, 600-800rpm) by moving the glass tube firmly up and down. After the homogenization, all buffer and foam was recollected and put into a 1.5ml Eppendorf tube. The homogenate was kept on ice until all other tissue samples were homogenized. Between to samples, homogenizer was cleaned with isolation buffer.

2. Fractionation procedure

All fractionation steps were carried out at 4°C. A summary of the procedure is shown on Fig. 2 for better understanding.

2.1. Preparing crude mitochondrial fraction from brain tissue

Homogenized samples were spun at 1300rcf (3700rpm) for 4 minutes. The supernatant was collected in an empty Eppendorf tube, while the pellet was resuspended in 750μl isolation buffer (with EGTA), then it was spun again with the same settings in order to release mitochondria from large cell debris. After the second spin, the supernatant was put together with the former supernatant collected from the first centrifugation step, and the pellet was discarded. The next step, the two supernatants collected in one tube are spun together at 13000rcf (11800rpm) for 11 minutes. The mitochondria containing pellet was saved, and

resuspended in 500µl isolation buffer. This stage is called “crude mitochondrial fraction” that still contains contaminating particles (cell organelles, myelin, cell debris, *etc*).

2.2. Percoll gradient fractionation procedure

For further purification, we used a simplified discontinuous Percoll gradient that merely consists of a 15% and a 0% Percoll layer (filtered Percoll stock solution is diluted to 15% with isolation buffer). Using a gradient centrifugation step, mitochondria and synaptosomes were separated from other, non-useful elements; on the other hand, easy enough to prepare even in a small-sized Eppendorf tube. The crude mitochondrial fraction was layered on 500µl of 15% Percoll solution in a special “Percoll tube” (2ml, conical shape). The Percoll gradient containing tubes were gently put into the centrifuge and spun at 22000rcf (15400rpm) for 7 minutes 40 seconds. In order to save the layers during the centrifugation, we used the lowest possible acceleration and the break was turned off. The two layers at the bottom (somal and synaptosomal mitochondria) were collected before the last steps by a fine pipette, while the top layer (cell membrane and myelin debris) was discarded.

Percoll, although considered as a harmless compound, has to be cleared off the sample. In order to obtain uninjured, coupled, viable mitochondria, the following cleaning steps were used before the final utilization: entirely filled tubes (of the resuspended sample) were spun at 22000rcf (15400rpm) for 11 minutes (full acceleration and break). After the centrifugation, the supernatant was carefully poured off. As the last step, the remaining, minimal amount of Percoll and the EGTA was removed by diluting it with 1ml of isolation buffer without EGTA. The tubes were centrifuged at 13000rcf (11800rpm) for 11 minutes, and the samples were stored as pellet in isolation buffer (without EGTA) on 4°C until the measurements.

3. Mitochondrial respiration rate measurements

Mitochondria containing fraction was transferred into respiration buffer and put into a Clark-type oxygen electrode chamber (Hansatech Instruments, Norfolk, UK) to measure their activity at 37°C. The electrode groove was filled with potassium chloride for establishing the electrode bridge between cathode and anode. Calibration was fulfilled by air saturated, deionized distilled water in order to establish the air line, while sodium dithionite for zero oxygen line. Using the protocol described by Toth et al [2], we measured the oxygen consumption by consecutively adding 5µl pyruvate together with 2.5µl malate, 2.5µl ADP, 1µl oligomycin and 2.5µl carbonylcyanide-4-(trifluoromethoxy)-phenylhydrazone to 50 µl of resuspended samples diluted with 450 µl respiration buffer in the electrode chamber.

The oxygen consumption was measured real time, and the results are expressed as consumed oxygen per minute (nmol O_2/ml). Five stages (each measured for 60 seconds) were distinguished according to the subsequently added respiration modifiers [3].

State 1 (St1): mitochondrial oxygen consumption in respiration buffer only, without the addition of any substrates that may affect mitochondrial activity. The measured mrr depends on the actual metabolic state of the mitochondria.

State 2 (St2): Mitochondrial function in the presence of oxidative substrates (pyruvate and malate in a final concentration of 5mM and 2.5mM, respectively) of the Krebs' cycle, but in lack of added substrate for the ATP synthase [4]. Under such conditions, the Krebs' cycle intensifies and oxygen consumption increases due to consequential facilitation of the terminal oxidation and oxidative phosphorylation if the down-regulating mechanisms are not active. Mitochondrial respiratory rate measured in St2 is limited by the amount of ADP present in the mitochondria at the time of sacrifice.

State 3 (St3): State 3 is initiated by adding ADP in a final concentration of 130μM. Being the substrate for ATP synthase, ADP is a major upregulator of mitochondrial respiration. Under such conditions, mrr increases if prior fuel supply of the hypothalamic tissue was sufficient. Therefore, if excess amount of ADP is added to the sample (Krebs' cycle is already fueled up), oxidative phosphorylation is limited exclusively by the activity of ATP synthase.

State 4 (St4): In state 4, oligomycin (2.5μM in the final concentration) is used to block the ATP synthase activity, therefore the oxidative phosphorylation, however the steps of terminal oxidation continues [5]. Under such conditions, oxygen consumption depends on the actual uncoupled stage and the activity of alternative oxidases of the mitochondria. Under physiological conditions, uncoupling and alternative oxidation play important roles in transient down-regulation of ATP biosynthesis when cellular energy needs drop. In case of fully viable mitochondria, oligomycin results in remarkably reduced mrr compared to that observed in state 3. It is to note that improper purification of the sample may lead easily to elevated state 4 mrr rendering a reliable evaluation unable.

State 5 (St5): At last FCCP [carbonylcyanide-4-(trifluoromethoxy)-phenylhydrazone]; is added to the sample in a final concentration of 5μM. FCCP is a cyanide derivative, therefore, by binding to and blocking cytochrome C oxidase, depletes all remaining oxygen from the sample (also acts as uncoupler, [6,7]). Decrease of oxygen level under such conditions depends on the initial (in vivo) metabolic state of the sampled tissue, and the amount of oxygen consumed during states 1-4 respiration. Therefore, this experimental setup is also known as total mitochondrial respiratory capacity.

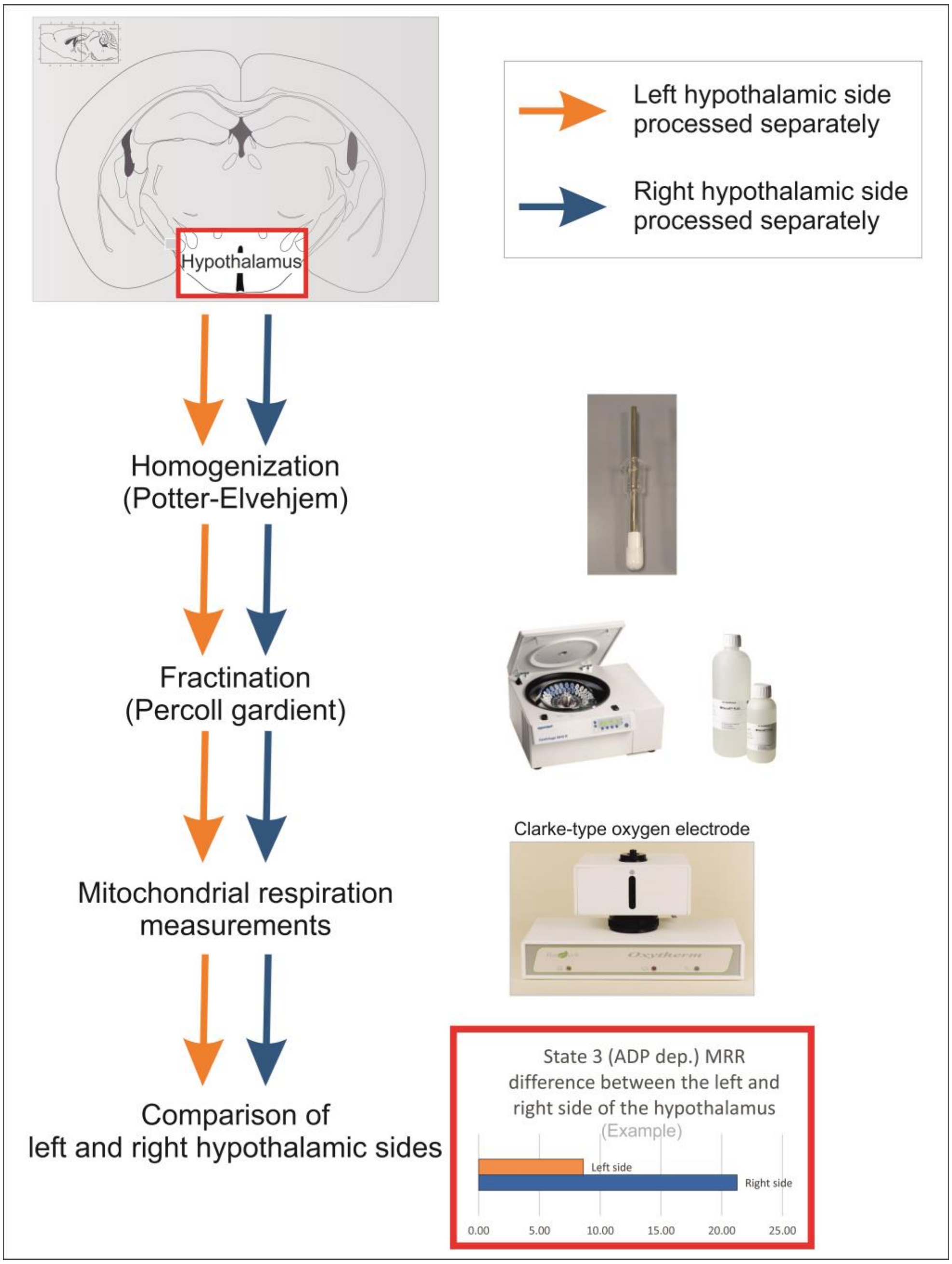

Fig. 1: Experimental design. Mitochondrial respiration rates (*mrr*) were measured on isolated hypothalamic synaptosomal and mitochondrial fractions in ovariectomized female rats. Left and right hypothalamic sides were homogenized, centrifuged, and measured separately.

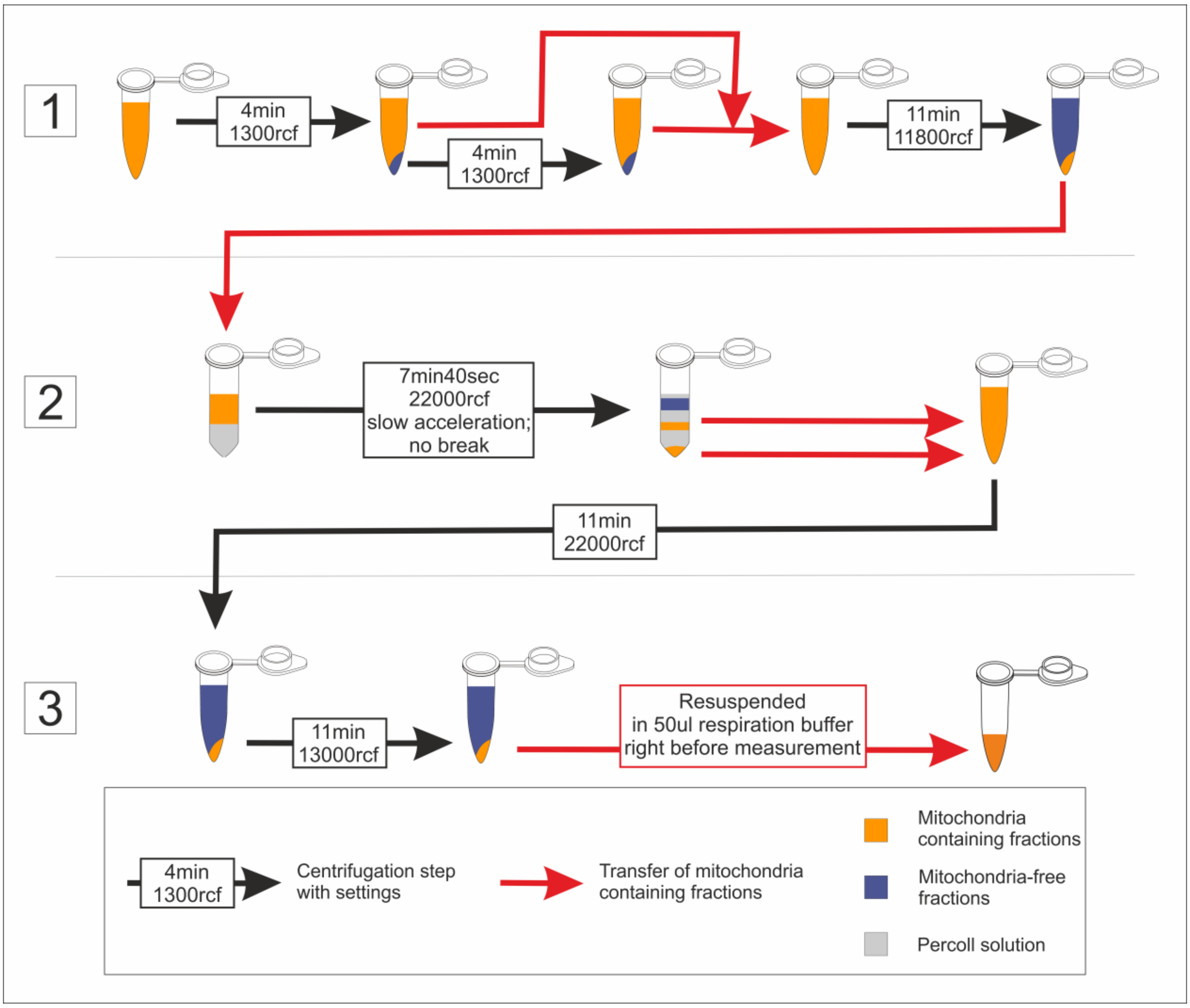

Fig. 2: Fractionation protocol for synaptosomal and non-synaptosomal mitochondria. 1) Preparation of so called ‘crude mitochondrial fration’. 2) Purification by Percoll gradient. 3) Clearing off the Percoll from the mitochondrial samples.

4. Cited articles in appendix

1. Grossmann A, Tsagarakis S, Korbonits M, Costa A. Determination of direct effects of cytokines on release of neuropeptides from rat hypothalamus by an in vitro method. In: De Souza EB, editor. Neurobiology of Cytokines: Methods in Neurosciences. 16th ed. New York: Academic Press; 2013. pp. 302–326.

2. Toth I, Kiss DS, Goszleth G, Bartha T, Frenyo L V, Naftolin F, et al. Hypothalamic Sidedness in Mitochondrial Metabolism: New Perspectives. Reprod Sci. 2014;21: 1492–8. doi:10.1177/1933719114530188

3. Brand MD, Nicholls DG. Assessing mitochondrial dysfunction in cells. Biochem J. 2011;435: 297–312. doi:10.1042/BJ20110162

4. Clark JB, Nicklas WJ. The metabolism of rat brain mitochondria. Preparation and characterization. J Biol Chem. 1970;245: 4724–31.

5. Dennis SG, Clark JB. The synthesis of glutamate by rat brain mitochondria. J Neurochem. 1978;31: 673–680.

6. Kalckar HM, Christopher CW, Ullrey D. Uncouplers of oxidative phosphorylation promote derepression of the hexose transport system in cultures of hamster cells. Proc Natl Acad Sci U S A. 1979;76: 6453–5.

7. Villa RF. Brain enzymes and ischemia. Eur Neurol. 1981;20: 245–52.
